# Treatment, pathological characteristics, and prognosis of pulmonary inflammatory myofibroblastic tumor–a retrospective study of 8 cases

**DOI:** 10.3389/fonc.2022.840886

**Published:** 2022-08-17

**Authors:** Xiao Zhu, Wen-Bang Chen, Fu-Bao Xing, Shao Zhou, Zhen Tang, Xiao-Jun Li, Lei Zhang, Yu-Chen Huang

**Affiliations:** ^1^ Department of Cardiothoracic Surgery, The First Affiliated Hospital of Bengbu Medical College, Bengbu, China; ^2^ Department of Pathology, The First Affiliated Hospital of Bengbu Medical College, Bengbu, China

**Keywords:** lung tumor, lung cancer, pathological characteristics, pulmonary inflammatory myofibroblastic tumor, prognosis

## Abstract

**Objective:**

Inflammatory myofibroblastic tumor (IMT) is a rare disease. We reviewed data from eight patients diagnosed with pulmonary IMT (PIMT) at our hospital with the aim of summarizing and analyzing the characteristics of PIMT to improve our understanding of the disease.

**Methods:**

From January 2012 to December 2019, eight patients underwent surgical intervention for PIMT at The First Affiliated Hospital of Bengbu Medical College. Resected tumors were subjected to pathological and immunohistochemical analyses. The follow-up duration for all patients ranged from 2 years and 3 months to 9 years and 9 months (median: 6 years and 9 months).

**Results:**

The male:female ratio was 5:3, and the mean age was 48.50 years (21–74 years). Two patients (25%) with lung disease discovered *via* chest computed tomography during physical examinations had not experienced any symptoms. Six patients (75%) presented at the hospital because of cough, expectoration, blood in sputum, and chest tightness. Lesions from all eight patients were surgically removed, and PIMT was confirmed based on pathological examinations and immunohistochemical results. No patient received additional treatment after discharge. All cases have been followed up to the time of writing, without any tumor recurrence or distant metastasis.

**Conclusion:**

The age of onset of PIMT is usually over 40 years, and its clinical symptoms are easily confused with those of lung cancer. PIMT can only be diagnosed by histopathology and immunohistochemistry. Complete surgical resection is the preferred treatment, as patients undergoing surgery require no additional treatment, such as chemotherapy, and the survival rate is good.

## Introduction

Inflammatory myofibroblastic tumor (IMT) is a rare disease. In 2020, the World Health Organization (WHO) identified IMT as a borderline tumor with potentially recurrent and rare metastatic properties (1). The most common site of IMT is the lung, although it can also occur in the abdomen, pelvis, head and neck, upper respiratory tract, limbs, lumbar tube, uterus, and other sites (2–6). To date, the etiology of IMT remains inconclusive, although recent studies have shown that IMT is associated with anaplastic lymphoma kinase (*ALK*) gene rearrangements, as well as overexpression of ALK protein, and the disease also involves fusion of genes such as *ROS1*, *NTRK3*, and *RET* (7–10). Furthermore, a fraction of IMT cases are associated with chromosomal abnormalities (11). The diagnosis of pulmonary IMT (PIMT) is rare and such cases account for only 0.04–0.7% of all lung masses (2, 12–14). Globally, there are very few studies related to PIMT. Therefore, to analyze and improve the understanding of the pathological characteristics, treatment modalities, and prognosis of PIMT, we aimed to review the data collected from a group of patients diagnosed with PIMT at our hospital and to review the literature related to such cases published in recent years.

## Material and methods

### Subjects

We reviewed the data of eight patients diagnosed with PIMT by histopathology and immunohistochemistry at The First Affiliated Hospital of Bengbu Medical College between January 2012 and December 2019, including information on clinical symptoms, treatment modalities, pathological features, and prognosis. Examinations conducted prior to surgery included laboratory examinations and computed tomography (CT) scans.In addition, to determine whether there is distant metastases of the tumor, we also performed cranial magnetic resonance imaging (MRI), bone emission computed tomography (ECT), and abdominal ultrasound on patients. None of these examination findings were abnormal.

### Histology and immunohistochemistry

After surgery, tumor specimens from each case were fixed with 10% neutral formaldehyde solution, extracted, paraffin-embedded, cut into 4-μm continuous sections, stained with hematoxylin and eosin (HE), and subjected to immunohistochemistry (EnVision; Agilent Technologies, Santa Clara, CA). The follow-up time ranged from 2 years and 3 months to 9 years and 9 months.

### Ethical approval

This study was conducted with approval from the Ethics Committee of The First Affiliated Hospital of Bengbu Medical College. Written informed consent was obtained from the participants.

## Results

**Figure 1:**
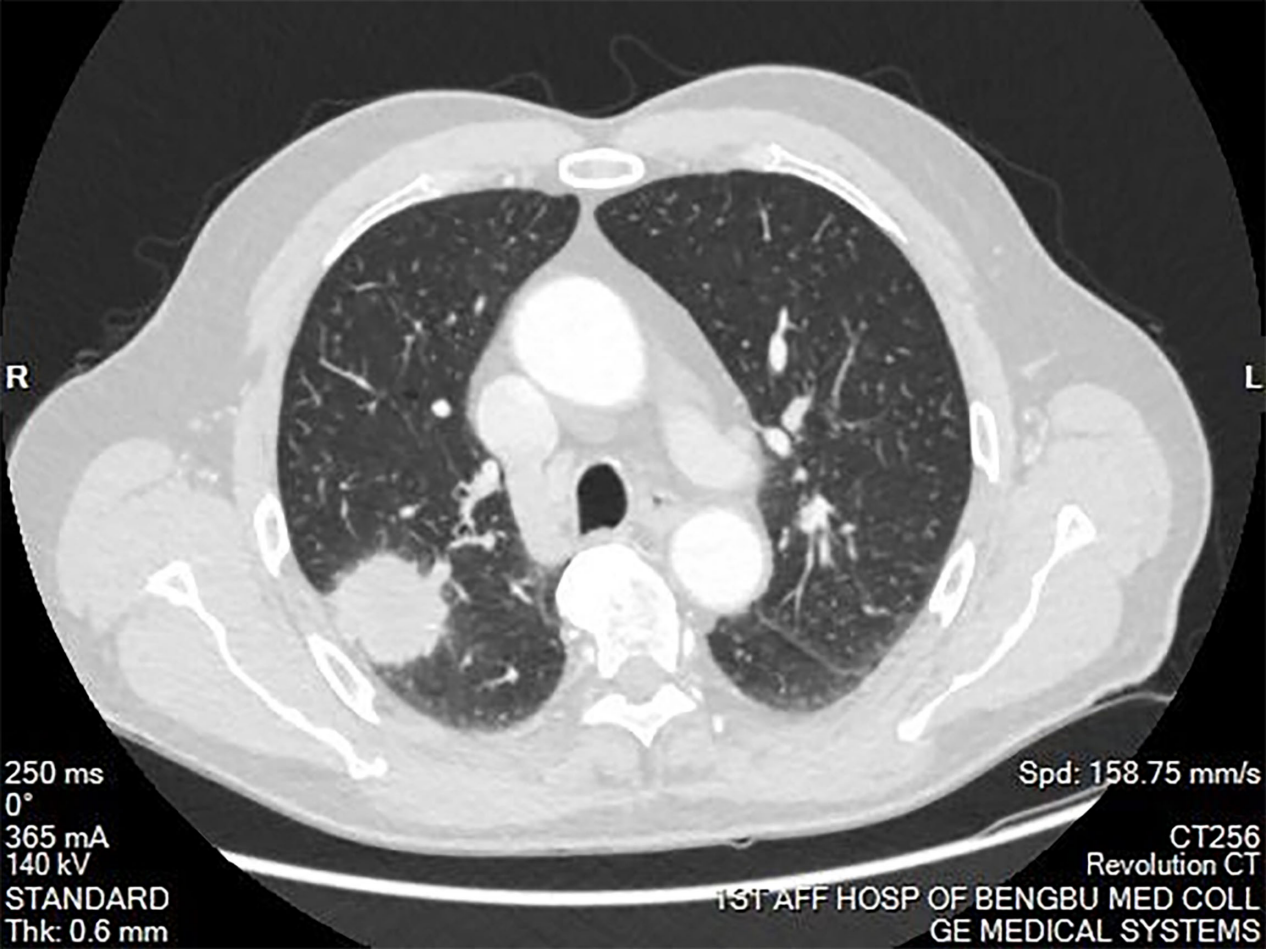
Computed tomography of the chest showing a mass in the right upper lobe of the lung. The mass was approximately 4cm in diameter. Burrs were present on the edge of the mass. A portion of the mass was connected to the pleura. The mass had uneven enhancement after the enhancement scan.

**Figure 2:**
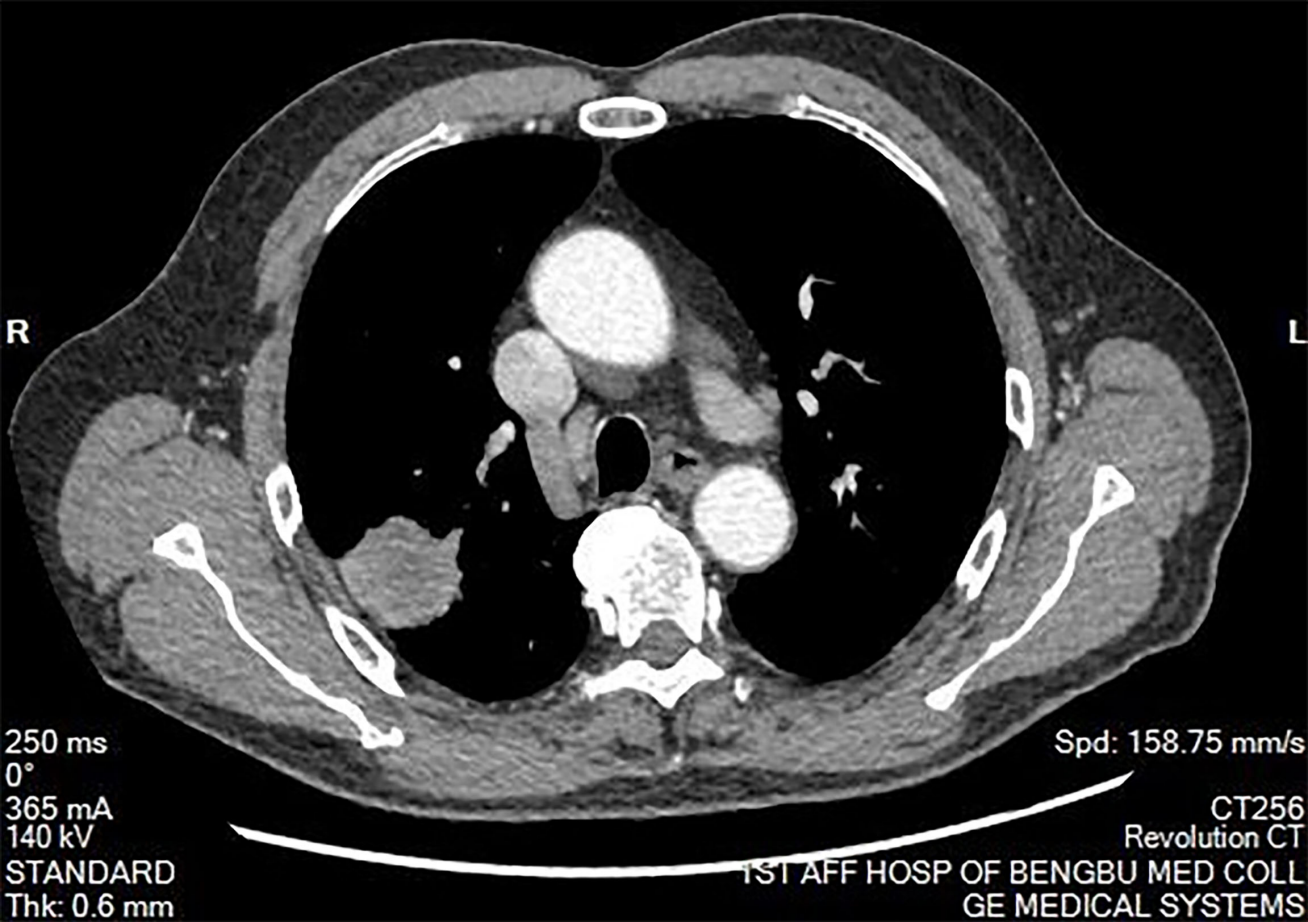
Mediastinal window of chest computed tomography. There was no abnormal mass in the mediastinum. The hilum on both sides is normal. There was no pleural effusion on both sides.

The clinical data of the eight patients included in the study are presented in **Table 1**. Among the eight patients, the male:female ratio was 5:3, and the mean age was 48.50 years (range: 21–74 years). Two patients (25%) with lung disease discovered by chest CT during a physical examination had experienced no symptoms. Six patients (75%) presented to the hospital because of cough, expectoration, blood in sputum, and chest tightness. Chest CT of the patients revealed a tumor diameter ranging from 2–4 cm (**Figures 1** and **2**). None of the patients had a long-term history of respiratory tract infections.

**Table 1:** Clinical data and immunohistochemical labeling of the eight patients.

Case	Sex/age (years)	Symptoms	Location/size	Surgery	Prognosis after surgery	Results of positive immunohistochemistry
1	M/38	Cough/ Hemoptysis	RLL/3.0 cm	Lobectomy	9 years 9 months, alive	ALK(++), Vim(+) SMA(+), Calponin(+), Ki67(+, 20~30%)
2	F/42	Cough/ Hemoptysis	RUL/2.0 cm	Lobectomy	9 years, alive	ALK(+), Vim(+), SMA(-), Calponin(-), Ki67(+, 10%)
3	F/46	None	RML/2.5 cm	Lobectomy	8 years, alive	ALK(+), Vim(+), SMA(+), Calponin(-), Ki67(+, 8%)
4	F/50	Cough/ Hemoptysis	RLL/4 cm	Lobectomy	7 years 7 months, alive	ALK(-), Vim(++), SMA(+), calponin(-), Ki67(-), CD68(-)
5	M/21	Cough/ Chest tightness	RUL/2.8 cm	Lobectomy	7 years 3 months, alive	ALK(+), Vim(++), SMA(+), calponin(-), Ki67(+, <5%)
6	M/50	None	LLL/2.3 cm	Lobectomy	6 years 7 months, alive	ALK(-), Vim(+++), SMA(-/+), calponin(-), Ki67(+/-, 10%)
7	M/67	Cough/ Chest tightness	LLL/3.0 cm	Lobectomy	4 years, alive	ALK(+), Vim(+), SMA(+), calponin(-), Ki67(-)
8	M/74	Thoracalgia	RUL/4.0 cm	Lobectomy+ lymph node dissection	2 years 3 months, alive	ALK(-), Vim(+++), SMA(+~++),calponin(-), Ki67(+, 20%)

F, female; M, male; RLL, right lower lobe; RUL, right upper lobe; RML, right middle lobe; LLL, left upper lobe; ALK, anaplastic lymphoma kinase; Vim, vimentin; SMA, smooth muscle actin.

A lobectomy was performed on all patients, resulting in successful tumor removal. None of the patients received additional treatment after discharge. There has been no tumor recurrence or distant metastasis in any patient to date.

**Figure 3:**
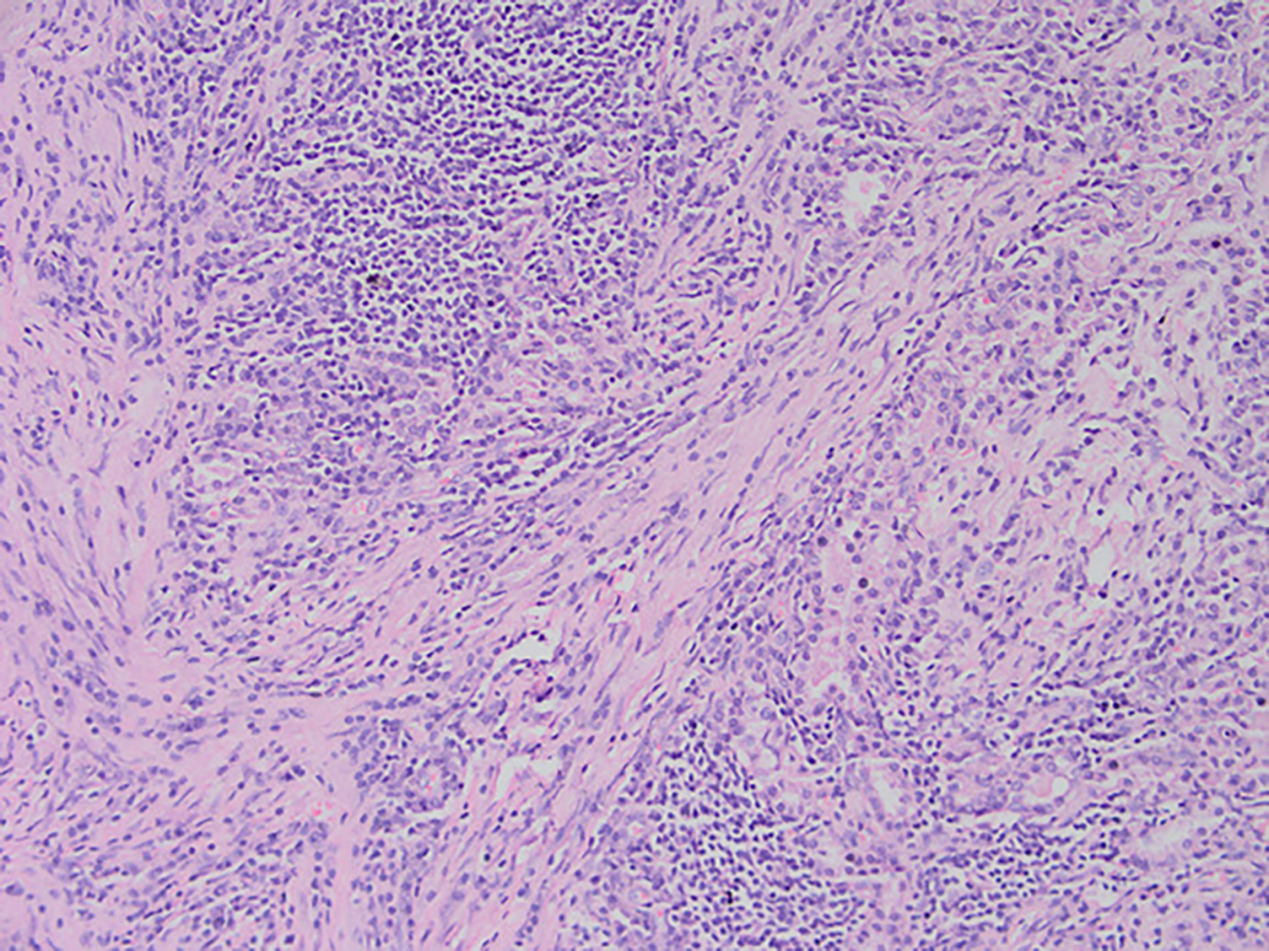
After staining of a pneumonia myofibroma, optical microscopy of the tumor reveals that it is composed of many spindle tumor cells, with some instances of plasma cell and lymphocyte infiltration into the stroma (magnification, ×200).

**Figure 4:**
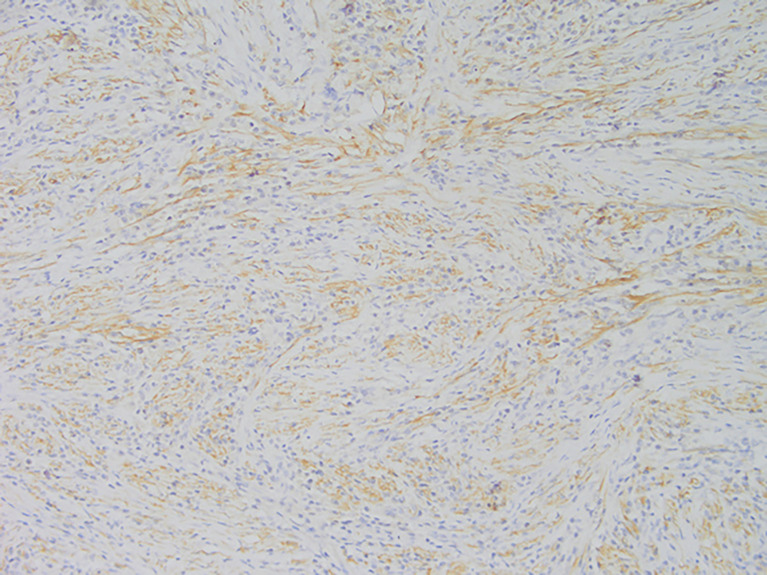
Representative image of anaplastic lymphoma kinase (ALK) expression (magnification, ×200).

**Figure 5:**
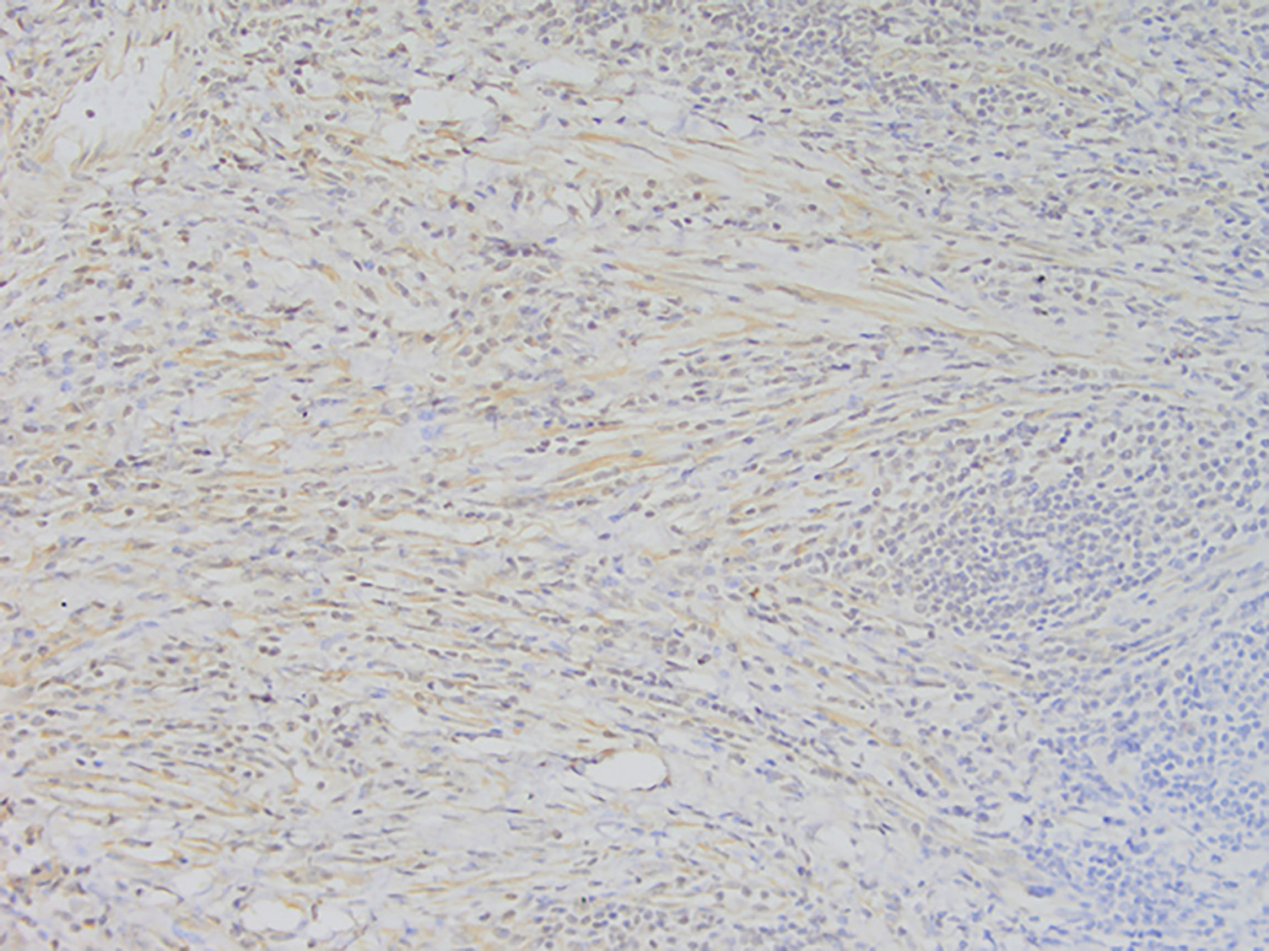
Representative image of vimentin expression(magnification, ×200).

**Figure 6:**
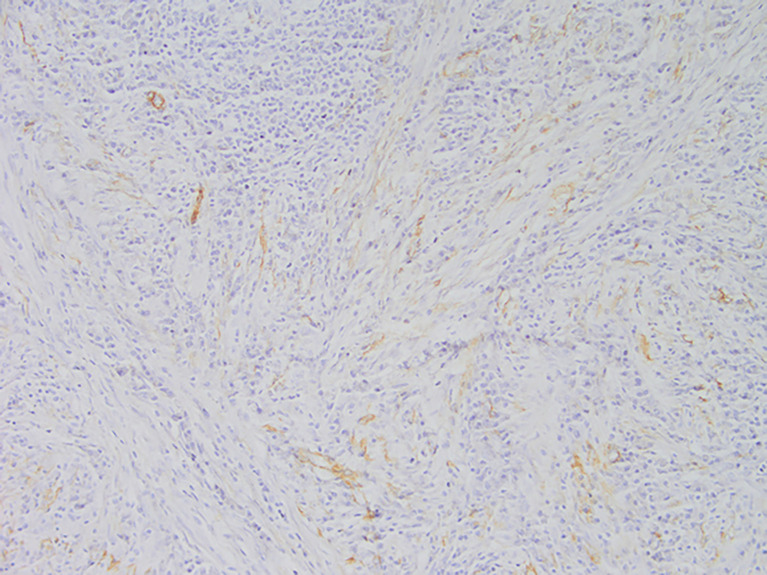
Representative image of smooth muscle actin (SMA) expression (magnification, ×200).

**Figure 7:**
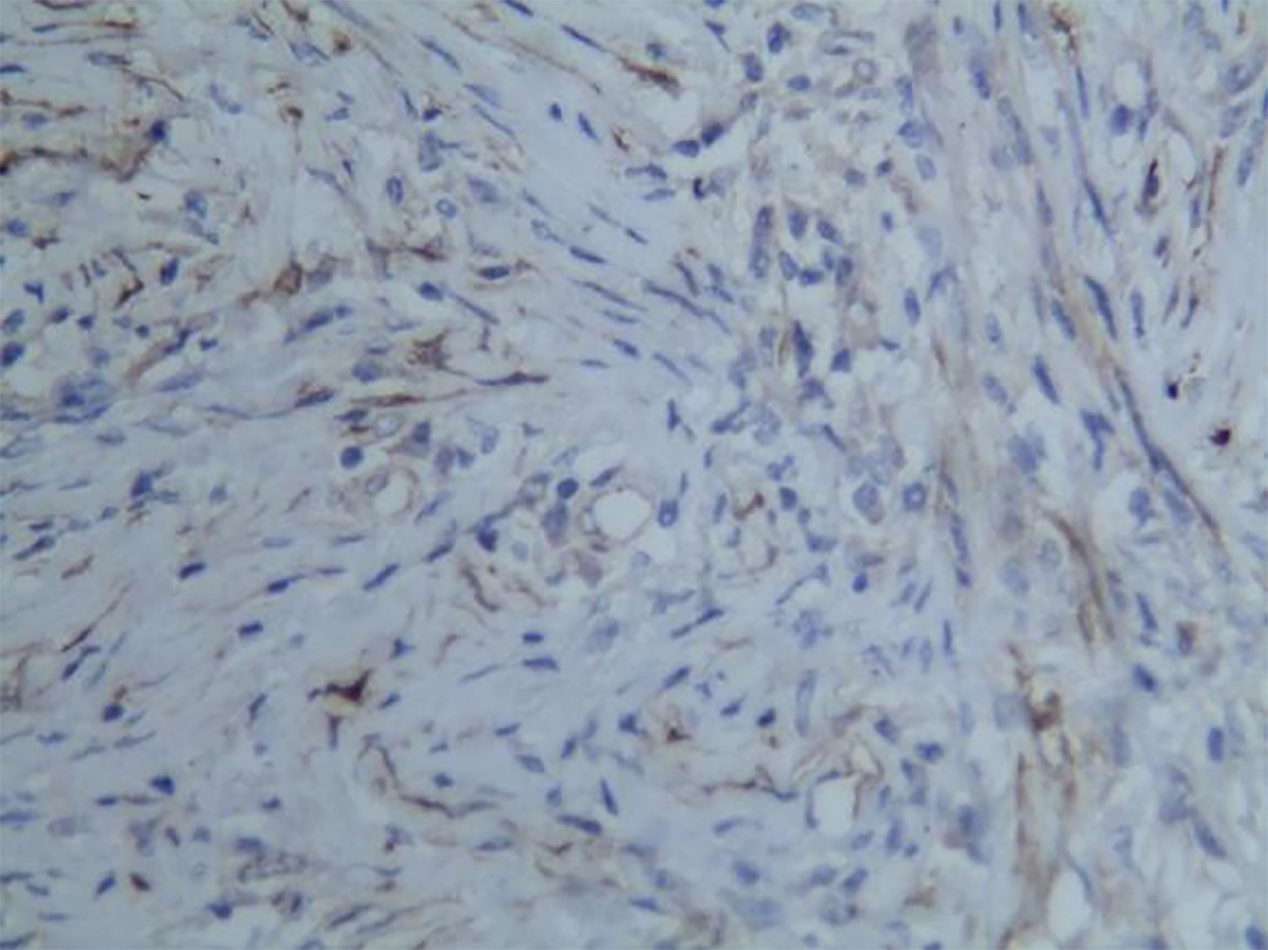
Representative image of calponin expression (magnification, ×200).

**Figure 8:**
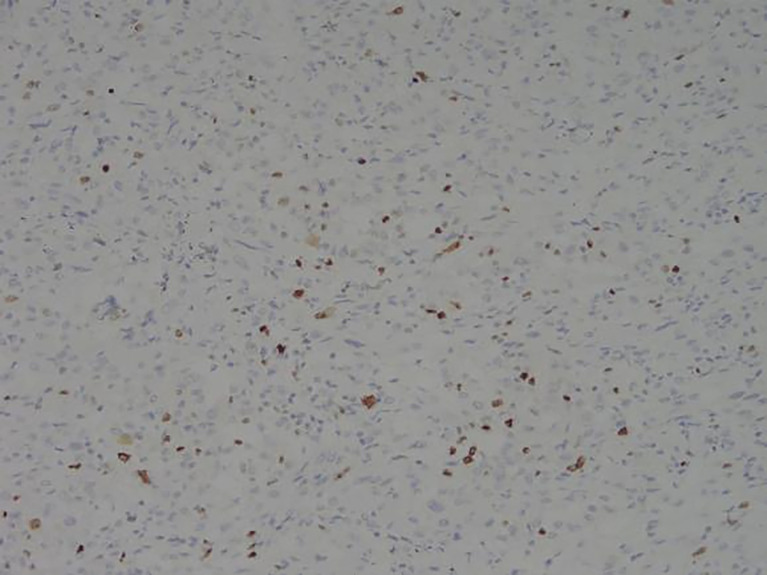
Representative image of Ki67 expression (magnification, ×200).

Among these patients, the largest tumor was 4 cm in diameter and the smallest was 2 cm in diameter, with an average of 2.95 cm. The tumors were mainly composed of spindle cells, with surrounding chronic inflammatory cell infiltration (**Figure 3**). In terms of immunohistochemical results, the following characteristics were observed: ALK labeling was positive in five cases (**Figure 4**), vimentin (VIM) labeling was positive in all eight cases (**Figure 5**), seven cases exhibited positive smooth muscle actin (SMA) labeling (**Figure 6**), one case demonstrated positive calponin labeling (**Figure 7**), and six cases showed positive Ki67 labeling (**Figure 8**). All eight patients were diagnosed with PIMT according to pathological features and immunohistochemistry results, and there was no lymph node metastasis.

## Discussion

IMT can occur at any age, although it most commonly occurs in young people, and there is no significant sex bias (2, 15–17). However, the mean age of our patients was 48.5 years, which is quite different from that reported in the literature. We believe this may be because the literature reports the mean age of patients with IMT at all body sites, whereas the present study only evaluated the characteristics of patients with PIMT specifically. In addition, in this study, there was no sex bias, which is consistent with previous reports.

The presence of cough, hemoptysis, and other symptoms reported by patients in this study support the view that the clinical manifestations of PIMT can be easily confused with lung cancer and tuberculosis (7). In X-ray and CT examinations, most cases of PIMT manifest as individual masses in the lung. The density inside the mass is basically uniform, and its boundaries tend to be clear, although lobulation and burr signs are occasionally seen (18), which is consistent with the CT characteristics of the patients in this study. Therefore, CT cannot distinguish PIMT from lung cancer. Although PIMT has also been reported in studies involving positron emission tomography (PET)/CT, spectral CT, magnetic resonance imaging (MRI), and other imaging methods (19, 20), imaging modalities alone cannot diagnose PIMT. Some scholars have reported that IMT has been misdiagnosed as lymphoma by PET/CT (21).

Pathology and immunohistochemistry are the most accurate methods for diagnosing IMT (20). Under the microscope, IMT is characterized by a series of myofibroblast proliferations and different types of inflammatory cell infiltration (14), which is consistent with the findings of this study. Most IMT immunohistochemistry has shown that spindle tumor cells were positively labeled by VIM, SMA, and ALK antibodies; more specifically, VIM labeling is usually strongly positive and diffusely observed in the cytoplasm of spindle cells, SMA labeling is mostly focal or diffusely positive, and ALK protein is expressed in 50–60% of cells (8). These immunohistochemical characteristics were also observed in the eight patients in this study. Therefore, we believe that VIM, SMA, and ALK are the three most important markers for the diagnosis of IMT by immunohistochemistry.

Although there are many ways to treat PIMT, surgical resection remains the first choice (14, 22). Although intraoperative frozen sections were collected in all cases, whether the tumors were benign or malignant could not be completely determined. At the same time, considering the clinical characteristics, CT results, and deep locations of the tumors, lobectomy was performed for all patients. In seven cases, the result of rapid frozen-section pathology was indicative of inflammatory myofibroblastoma. Therefore, only lobectomy was performed. In one case, the result of rapid frozen-section pathology was indicative of IMT, but the possibility of a malignant tumor was not ruled out, so a lobectomy with lymph node dissection was performed. Casanova and colleagues (23) believe that the prognosis of patients with PIMT who undergo early surgery is usually ideal, and there is no need for adjuvant treatment such as chemotherapy. Our results are consistent with this assertion, and none of the patients received additional treatment after discharge. To date, there have been no recurrences or distant metastases.

We performed lymph node dissection in one patient and the result was negative. There is no definitive conclusion regarding whether lymph node dissection should be conducted in patients with PIMT. Some researchers have found the existence of cancer stem cells in PIMT tissues (24), and some studies have reported that the lymph nodes removed during surgery in patients with PIMT were positive (23). In addition, cervical lymph node metastasis has occurred 3.5 years post-operation (25). Moreover, the WHO points out that IMT is a borderline tumor with the potential for recurrence and rare metastasis. Based on the above aspects, we suggest that lymph node dissection should be performed if the pathological results of intraoperative frozen sectioning suggest PIMT. However, the current sample size is small, and there is still a lack of research in this field. More studies must be conducted in the future to verify the necessity of lymph node dissection in these patients.

The prognosis of patients with PIMT is good (22, 23), with a 5-year survival rate of 91.3% and a 10-year survival rate of 77% (2). Studies have found that all metastatic IMTs are ALK-negative, and ALK positivity may be a good prognostic indicator of IMT (26). However, the latest research by Casanova and colleagues (23) shows that even patients who cannot undergo surgery and those who are ALK-negative have a good prognosis. Regardless of whether ALK labeling was positive or not, the eight patients whose data we reviewed experienced no recurrence or metastasis after surgery, which was consistent with the results of Casanova et al. (23). Some patients with IMT who have tumor tissue removed still experience relapse and distant metastasis (25–27). In one study, researchers followed up 23 patients after PIMT excision for 2–127 months; in those cases, recurrence only occurred twice after operation, and there was no recurrence after reoperation (28). If only local recurrence occurs and the patient’s physical condition is good, reoperation is still recommended (7). Systemic therapy is reserved for patients with unresectable, progressive or metastatic disease and whose body is unable to withstand lobectomy. There is controversy regarding the treatment of PIMT with steroids. On the one hand, as early as 1991, the treatment of PIMT with steroids was reported (29), and on the other hand there are reports that steroids may have an enhancing effect on IMT cell proliferation (30). In addition, there are also some reports on non-steroidal anti-inflammatory drugs(NSAID) treatment for PIMT that are ALK-negative (31). Chemotherapy is a valid option for advanced IMT (23). One study has confirmed that Anthracycline-based and methotrexate plus/minus vinorelbine/vinblastine (MTX-V) regimens are very effective in IMT (17). Studies have reported the cases of using radiotherapy for recurrence of surgical resection (25), but currently there is no sufficient evidence to prove the efficacy of radiotherapy. There are reports that gene-targeted drugs are used to treat IMT (32–40). The US National Comprehensive Cancer Network recommends the use of crizotinib as the standard of care for IMT with ALK-positive (36). Ceritinib, a second-generation ALK inhibitor, has also been shown to be effective in IMT (41).

## Conclusion

PIMT is a rare tumor type. Due to the lack of specificity in clinical and imaging manifestations, the diagnosis of PIMT can only be made based on pathology and immunohistochemistry results. Complete surgical resection is the preferred treatment in such cases and usually results in satisfactory outcomes. Because local recurrence and metastasis is possible in some cases of IMT, we recommend close, long-term follow-up. Because the disease is rare and the sample size of this study is small, the views described in this paper need to be supported by more research, such as multicenter and large-sample studies to provide more concrete clinical recommendations.

## Data availability statement

The original contributions presented in the study are included in the article/supplementary material. Further inquiries can be directed to the corresponding author.

## Ethics statement

The studies involving human participants were reviewed and approved by Ethics Committee of The First Affiliated Hospital of Bengbu Medical College. The patients/participants provided their written informed consent to participate in this study.

## Author contributions

XZ, WBC and LZ performed the surgeries, reviewed the literature, and contributed to manuscript drafting; FBX, SZ, and ZT reviewed the literature and contributed to manuscript drafting; and XJL and YCH were responsible for the revision of the manuscript for important intellectual content. All authors issued final approval for the version to be submitted.

## Funding

The study was supported by the Scientific Research Foundation of Education Department of Anhui Province of China (KJ2019A0340).

## Conflict of interest

The authors declare that the research was conducted in the absence of any commercial or financial relationships that could be construed as a potential conflict of interest.

## Publisher’s note

All claims expressed in this article are solely those of the authors and do not necessarily represent those of their affiliated organizations, or those of the publisher, the editors and the reviewers. Any product that may be evaluated in this article, or claim that may be made by its manufacturer, is not guaranteed or endorsed by the publisher.
